# Preparation and Properties of Electrodeposited Ni-B-Graphene Oxide Composite Coatings

**DOI:** 10.3390/ma15062287

**Published:** 2022-03-20

**Authors:** Desen Cheng, Lan Zhang, Yongchao Zhu, Huimin Xia, Na Li, Wentao Song, Hui Bai, Huizhong Ma

**Affiliations:** 1 School of Mechanics and Safety Engineering, Zhengzhou University, Zhengzhou 450001, China; cheng.ds@icloud.com (D.C.); a13523006777@gs.zzu.edu.cn (H.X.); zzulina@zzu.edu.cn (N.L.); aitaotao@gs.zzu.edu.cn (W.S.); 202022252014806@stu.zzu.edu.cn (H.B.); 2 Department of Railway Engineering, Zhengzhou Railway Vocational and Technical College, Zhengzhou 451460, China; zhuyongchao@zzrvtc.edu.cn; 3 Center of Advanced Analysis and Gene Sequencing, Zhengzhou University, Zhengzhou 450001, China

**Keywords:** graphene oxide, Ni-B-GO, electrodeposition, properties, strengthening mechanism

## Abstract

With the rapid development of modern industries, the surface quality and performance of metals need to be improved. Composite electrodeposition (co-deposition) has evolved as an important technique for improving the surface performance of metal materials. Herein, a new type of graphene oxide (GO)-reinforced nickel–boron (Ni-B) composite coating was successfully prepared on a 7075 aluminum (Al) alloy by co-deposition. Characterization revealed a significant improvement in the mechanical and anti-corrosion properties of the composite with the incorporation of GOs. The composite showed a rougher, compact, cauliflower-like morphology with finer grains, a higher hardness (1532 HV), a lower rate of wear (5.20 × 10^−5^ mm^3^∙N^−1^∙m^−1^), and a lower corrosion rate (33.66 × 10^−3^ mm∙y^−1^) compared with the Ni-B alloy deposit (878 HV, 9.64 × 10^−5^ mm^3^∙N^−1^∙m^−1^, and 116.64 × 10^−3^ mm∙y^−1^, respectively). The mechanism by which GOs strengthen the Ni-B matrix is discussed.

## 1. Introduction

Today’s society is facing great pressure in terms of energy conservation and emission reduction. Weight reduction in the aerospace, automobile, and marine industries has become a primary trend to relieve this pressure. There is now a palpable desire to expand the application of lightweight materials such as aluminum (Al) alloys to reduce energy bills and maintenance costs [1]. Al alloys have attracted much attention for use in transportation, packaging, and architecture due to their good properties, such as low density, high specific strength, and excellent machinability [2,3]. However, with advances in the preparation of advanced materials, the poor surface performance of Al alloys is gradually being exposed. The poor wear and corrosion resistance of Al alloys not only necessitate the use of a great deal of resources and impose huge maintenance costs but also limit their further application in modern industry [3,4]. Different from the improvement of an entire material using power metallurgy, electrodeposition offers a more economical and practical approach to resolving this problem by enhancing the surface properties of metal materials, since most failures occur on the surface or subsurface [5].

Hard chromium (Cr) plating has been extensively used as a proactive film for various components and parts since the technology was invented. However, hexavalent chromium (Cr^6+^) produced in the preparation of hard Cr coating has disadvantages such as being toxic and creating environmental pollution [6,7]. Therefore, the international community is gradually banning Cr^6+^-plating technology while developing alternative technologies. As a promising environmentally friendly alternative to Cr^6+^ coating, Ni-B-alloy coating has garnered significant attention for its outstanding mechanical and anti-corrosion properties [8,9,10,11]. Until recently, the urgent demand for high-performance materials has forced researchers to conduct more research on Ni-B coatings. A number of studies have reported that the incorporation of various micro- and nanoparticles such as Al_2_O_3_ [12], SiC [13,14], ZrO_2_ [15,16], TiN [17,18], AlN [19,20], TiO_2_ [21], and diamond [22] within the Ni-B matrix can further enhance the mechanical and anti-corrosion properties, among others. The embedding of these solid particles in a Ni-B alloy coating provides longer-lasting and effective protection for the surfaces of various mechanical components working in extreme environments such as high-load, high-temperature, or corrosive environments.

Graphene, a honeycomb-like sheet of carbon that is only one atom thick, has attracted much attention for several years due to its special configuration and capability, and it is considered a promising reinforcing particle [23,24,25,26]. However, the homogeneous dispersion of graphene in electrolytes and the electrodeposition of high-performance composite coatings remains a technical challenge due to the high active surface area [27]. Therefore, graphene is not suitable for direct addition into a bath solution. Graphene oxide (GO), the product of the chemical oxidation and stripping of graphene, has garnered considerable attention. Many oxygen-containing functional groups can improve the ability of GO to disperse in an electroplating bath, allowing the fabrication of a uniform deposit [28]. In the reported studies, some researchers have used electroless plating to carry out the co-deposition of GO within a Ni-B matrix on a steel substrate [29,30]. Han et al. presented detailed information about the electroless plating process for the preparation of a Ni-B-GO coating on the surface of SiC/Al composites but did not conduct an in-depth study of the characteristics and properties of the composite coating [31]. Duru et al. prepared Ni-B-GO coatings on a St37 steel substrate using the pulsed electrodeposition technique and investigated the microstructure and properties of the coatings [32]. Al is the second important metal and plays an irreplaceable role in the field of advanced manufacturing. However, there is little information about the electrodeposition, microstructural characterization, and surface properties of Ni-B-GO coatings on Al alloy substrates.

This work intended to obtain an ideal Ni-B-GO coating on 7075 Al alloy, embedding GOs as the reinforcing particles by composite electrodeposition in a Ni-B-electroplating bath. In addition, the effect of GOs on the surface morphology, component, mechanical, and anti-corrosion properties of the Ni-B-GO composite coatings were investigated and are discussed.

## 2. Materials and Methods

Ni-B-GO composite was coated on the surface of the Al alloy using direct current co-deposition in the Ni-B electrolyte containing 0.15 g∙L^−1^ GOs. GOs were obtained from the Chengdu Institute of Organic Chemicals Co., Ltd. using a refined Hummers method. The size of the GOs used in the experiments was about 8–15 μm. The composition of the electrolyte and the experimental parameters are presented in Table 1. All the chemical reagents used for preparing the Ni-B-plating solution were purchased from Sinopharm Chemical Reagent Co., Ltd. in Shanghai, China. A pure Ni plate was used as the soluble anode to replenish the metal ions for the electrolyte. Some 7075 Al alloy sheets with sizes of 25 mm × 25 mm × 3 mm acted as the cathode to provide reaction sites for the reduction of Ni-B ions. Before electrodeposition, abrasive papers (240–1000 meshes) and silica sand (280 meshes) were successively used for polishing and sandblasting the substrate. Then, the Al alloy samples were immersed in NaOH (3%) and H_2_SO_4_ (3%) for 1 min, followed by ultrasonic cleaning in deionized water to form a clean deposition surface. In addition, the electrolyte with GOs was treated using an electric mixer at 500 r∙min^−1^ for 30 min and then ultrasonicated at room temperature for 2 h to improve the dispersion of the GOs in the bath solution.

Scanning electron microscopy (SEM, Helios G4 CX) with an energy-dispersive spectrometer (EDS) was utilized to investigate the morphology as well as the chemical components of the coatings. The Raman spectra of the GOs were acquired using a Raman spectrometer (LabRAM HR Evo) with a wavelength of 532 nm. Automated surface area and pore size analyzer (ASAP2460, America) was used to measure BET (Brunauer-Emmett-Teller) surface area of GOs. Temperature and Time of degasification are 100 °C and 6 h. X-ray diffractometry (XRD, Empyrean) with a Cu Kα radiation of λ = 0.154 nm was applied to analyze the phase structure of the coatings in the range of 30°–85°. The grain size was computed using the Scherrer equation [34,35]:

(1) D=K γ/βcosθ

where *D* is the average grain size (nm), *K* is the Scherrer constant (0.89), *γ* is the diffracted wavelength (0.154 nm), *β* is the full width at half maximum (rad), and *θ* is the diffraction angle (°). The preferred orientations of the coatings were estimated according to Equation (2) [36]:

(2) $$T_{c(h\;k\;l)}=\frac{I_{\left(h\;k\;l\right)}}{\sum I_{\left(h\;k\;l\right)}}\;\times \;\frac{\sum I_{0(h\;k\;l)}}{I_{0(h\;k\;l)}}\;\times \;100\%$$

where *T*_c_ represents the texture coefficient, (h k l) are the indices of the crystal face, and *I* is the intensity of the diffraction peak; the subscript 0 refers to the standard sample.

A Vickers microhardness tester (HXD-1000TM) was applied to evaluate the surface hardness at a load of 0.98 N with a dwell time of 15 s. Five measurements of every sample were averaged to obtain the valid value. Every measurement was repeated at least twice to ensure the repeatability of the experiments and the validity of the data. The tribological properties of the samples were evaluated using an MS-T3000 rotary friction tester, and a ZrO_2_ ball (Φ4 mm) loaded with 300 gf acted as the counterpart, sliding on the surfaces of the samples for 30 min at a speed of 200 r min^−1^. The morphology and elemental distribution of the wear surface were studied by SEM and EDS, respectively. Additionally, an MFT-4000 material surface tester was used to measure the wear rate and surface roughness of the coatings.

An electrochemical workstation (CHI 760E) coupled with a three-electrode cell was used to investigate the corrosion resistance of the substrate and coatings. A platinum sheet and saturated calomel electrode (SCE) were applied to act as the counter electrode and reference electrode, respectively. The as-prepared samples, with an exposed area of 1 cm^2^, served as the working electrode. All the measurements regarding corrosion behavior in this study were performed in a neutral NaCl solution (3.5 wt%) at room temperature. The corrosion current (*i*_corr_) was calculated using the method of Tafel extrapolation from the potentiodynamic polarization (PDP) curves recorded at a scan rate of 1 mV∙s^−1^. The corrosion rate (*CR*) can be obtained using the following formula: ($CR=3.27\times 10^{-3}i_{\mathrm{corr}}E.W./\rho$) [37], where *E.W.* refers to the electro-chemical equivalent and *ρ* represents the material density. In addition, electrochemical impedance spectroscopy (EIS) tests were carried out at the stable open-circuit potential (OCP) in a range of frequencies from 100 kHz to 0.1 Hz.

**Figure 1:**
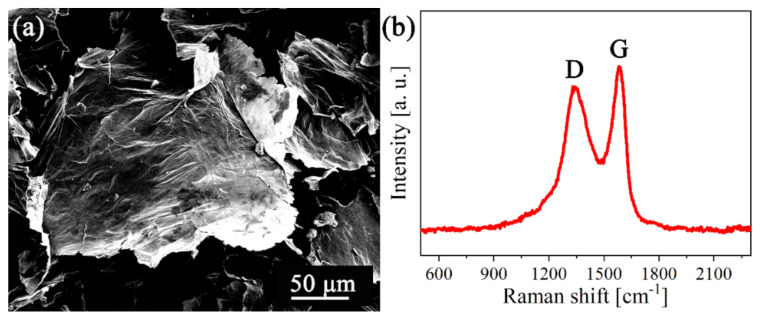
(**a**) Morphology and (**b**) Raman spectrum of GOs.

The SEM image of the GOs shown in Figure 1a suggests a highly crumpled surface for these nanosheets. As shown in Figure 1b, two characteristic peaks at 1345 and 1582 cm^−1^ were found in the Raman spectrum, corresponding to the D and G peaks of GO, respectively. The high intensity ratio value of D/G (0.89) confirmed that there are many defects on the surfaces of GOs [36]. BET surface area of GOs is 34.99 m^2^∙g^−1^.

## 3. Results and Discussion

### 3.1. Morphology and Composition

**Figure 2:**
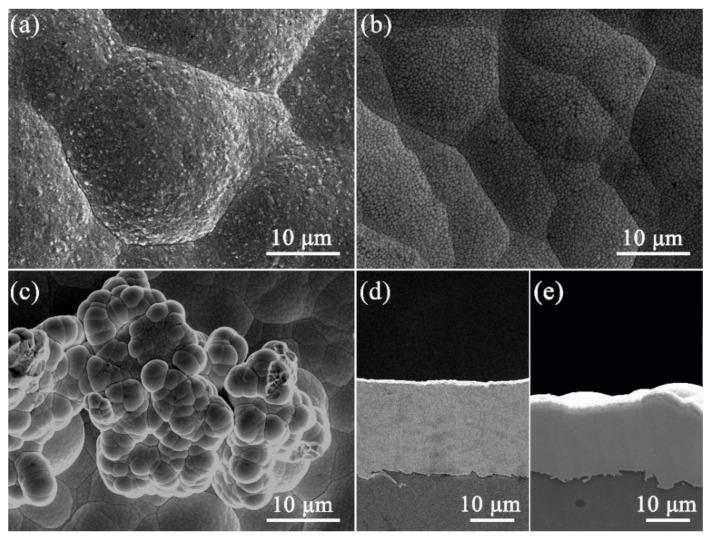
The surface morphologies of (**a**) pure Ni, (**b**) Ni-B, and (**c**) Ni-B-GO. The cross sections of (**d**) Ni-B and (**e**) Ni-B-GO.

In order to study the influence of the addition of B on the microstructure of the Ni-B alloy, we also prepared a pure Ni coating under the same parameters to contrast with the Ni-B and Ni-B-GO coatings. Figure 2 shows the surface morphology of the deposits. Different from the pyramidal crystalline structure of the pure Ni coating shown in Figure 2a, the Ni-B coating demonstrated in Figure 2b showed a uniform and fine surface with a flattened cellular structure. This change could be attributed to the alloy phase with different microstructures and grain refinement caused by the incorporation of B [4]. This phenomenon matched well with that in the existing literatures [21,38].

With the addition of GOs, there were obvious changes in the surface morphology of the Ni-B-GO coating, as shown in Figure 2c. The composite exhibited a rougher surface consisting of some cauliflower-like asperities formed from the spherical growth of the Ni-B matrix. GO, as a graphene derivative, provided plentiful active nucleation sites for the crystallization of the Ni-B matrix due to its superior electrical conductivity and huge specific surface area, meaning that Ni-B atoms were preferentially nucleated around the nanoplatelets. In addition, the presence of these nodules increased the surface roughness from 1.22 to 2.82 μm.

**Figure 3:**
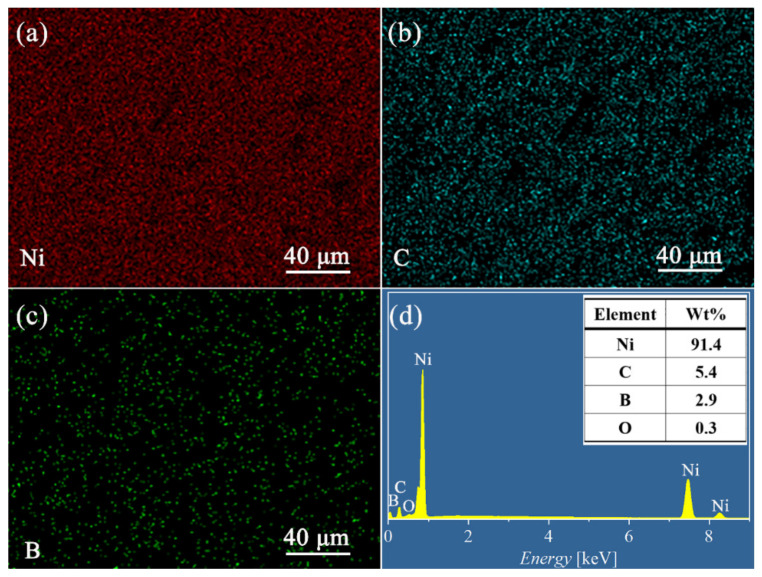
(**a–c**) EDS mapping and (**d**) spectra of the Ni-B-GO coating.

The cross sections of the Ni-B and Ni-B-GO coatings are presented in Figure 2d,e, respectively, showing a clear interface without any detectable interspace or delamination between the substrate and the films. The thicknesses of the Ni-B and Ni-B-GO coatings were 19.5 and 16.9 μm, respectively. Figure 3 shows the EDS mapping and spectra of the Ni-B-GO coating, and the Ni, B, and C elements were shown to be evenly distributed throughout the test area. The wt% values of the B in the Ni-B and Ni-B-GO coatings were determined to be 3.1% and 2.9%, respectively. Therefore, the incorporation of GOs had a limited effect on the reduction reaction for B ions. As the samples were rinsed under ultrasonication after plating, no obvious GOs were detected on the composite surface. However, the EDS spectra of the Ni-B-GO coating displayed in Figure 3d showed that the components were Ni (91.4 wt%), C (5.4 wt%), B (2.9 wt%), and O (0.3 wt%), confirming the successful embedding of GOs into the composite film.

### 3.2. Phase Compositions

**Figure 4:**
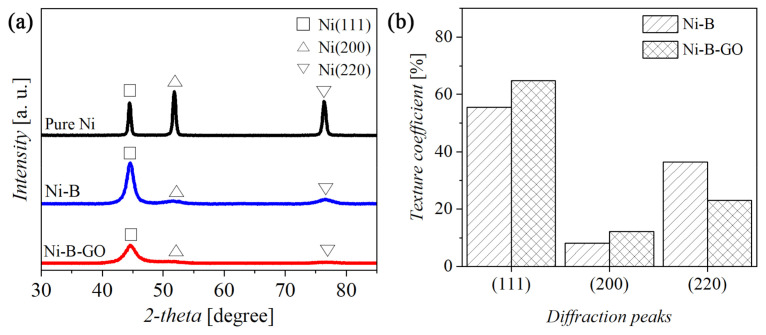
(**a**) XRD patterns and (**b**) texture coefficient of the coatings.

Figure 4 reveals the X-ray patterns of the coatings as well as the texture coefficient (*T*_c_). As clearly shown in Figure 4a, both Ni-B and Ni-B-GO had three characteristic peaks at the crystal faces (111), (200), and (220), similar to those of the pure Ni coating. In addition, the diffraction peaks of the (111) crystal faces for the alloy (44.58°) and the composite coating (44.62°) shifted slightly to the higher 2*θ* in comparison to the Ni coating (44.46°). This phenomenon can mainly be attributed to the fact that the Ni-B alloy obtained by the electrodeposition consisted of the mixed substituted-interstitial-type solid solution. B atoms with a small atomic radius occupy the Ni-lattice sites or the gaps between the Ni atoms [39,40]. With the addition of TMAB in the electrolyte, the incorporation of B atoms into the structure resulted in the generation of the preferred orientation at [111] and reduced the average grain size from 19.7 (Ni coating) to 5.2 nm [41,42]. Figure 4b shows the *T*_c_ of the Ni-B and Ni-B-GO coatings. Compared with the Ni-B coating, the most apparent change for the Ni-B-GO coating was the broadening of the (111) peak and the enhancement of the extent of the preferred orientation. The promoting effect of GOs on nucleation during electrodeposition further refined the grain size of the composite (2.7 nm) [43]. However, owing to the low amount of the nanoparticles, no obvious C peaks corresponding to GOs were detected in the XRD pattern of the composite.

### 3.3. Microhardness

**Figure 5:**
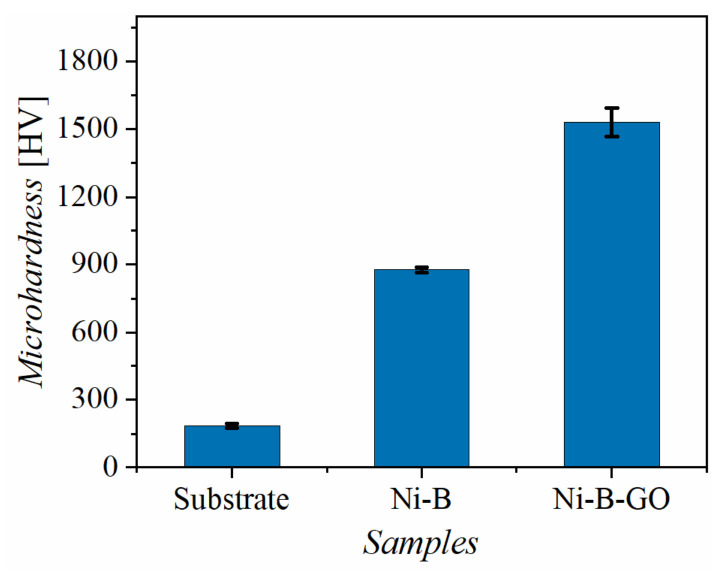
Microhardness of the substrate, Ni-B coating, and Ni-B-GO coating.

The Vickers hardness values of the substrate, Ni-B alloy coating, and Ni-B-GO composite coating are shown in Figure 5. Microhardness tests revealed that the Ni-B-GO coating had the highest hardness value (1532 HV), which was 828% and 174% of the values for the substrate (185 HV) and the alloy coating (878 HV), respectively. The grain refinement of the composite and the strengthening effect of GOs were responsible for the improvement of the microhardness. The Hall–Petch formula revealed that more grain boundaries formed from grain refinement can improve the surface hardness by strengthening the resistance to dislocation motion [38,44]. GO, as a typical graphene derivative, has a huge specific surface area and unique mechanical properties that contribute to sharing the external force applied to the Ni-B matrix by transferring the interfacial load [34,38].

### 3.4. Tribological Properties

**Figure 6:**
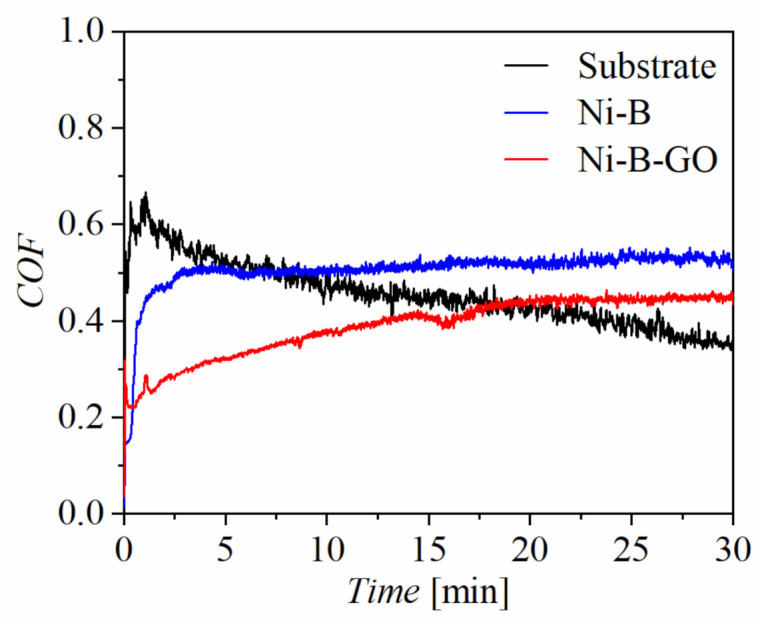
COF curves of the substrate, Ni-B coating, and Ni-B-GO coating.

Figure 6 shows the relationship between the coefficient of friction (COF) and test time for the substrate, Ni-B coating, and Ni-B-GO coating. The COF of the composite increased slowly and gradually tended to be stable, while that of the Ni-B coating rose rapidly in a short period time and maintained a high value. With the addition of GOs in the electrolyte, the average COF of the Ni-B-GO coating (0.39) was reduced in comparison to that of the substrate (0.46) and the Ni-B coating (0.51).

Compared with the Al alloy (2.16 × 10^−3^ mm^3^∙N^−1^∙m^−1^) and the Ni-B coating (9.64 × 10^−5^ mm^3^∙N^−1^∙m^−1^), the lowest wear rate of the Ni-B-GO composite coating (5.20 × 10^−5^ mm^3^∙N^−1^∙m^−1^) amply demonstrated the strengthening effect of GOs on the wear resistance. The Archard model indicates that the surface hardness is positively correlated with the wear resistance [27]. The higher hardness played an important role in the good wear resistance of the composite. The weak shear force between the GO layers endowed it with good self-lubricity. When the nanoparticles were brought into the friction interface during the wear process, the interformational sliding of the GOs alleviated direct contact between the friction pairs, which contributed to the improvement in the tribological properties of the Ni-B-GO deposit [4,27].

**Figure 7:**
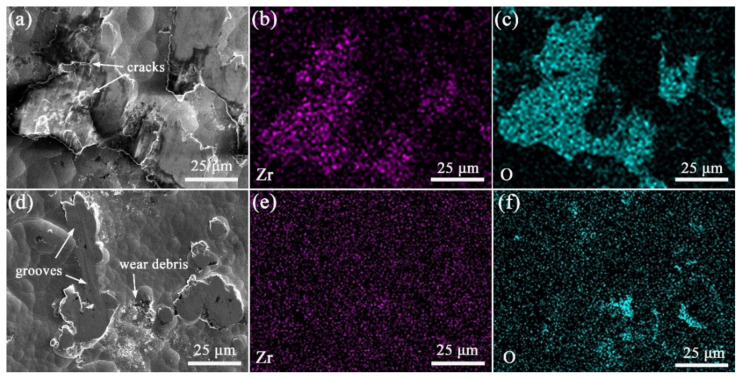
SEM images and EDS mapping of worn surface for (**a**–**c**) Ni-B alloy and (**d**–**f**) Ni-B-GO composite coating.

**Figure 8:**
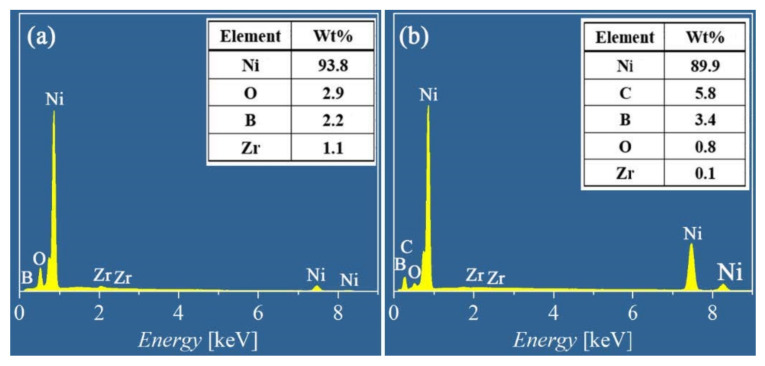
EDS spectra of worn surface for (**a**) Ni-B alloy and (**b**) Ni-B-GO composite coating.

Figure 7 shows the worn morphology and EDS mapping of the alloy and composite coatings. Heavy plastic deformation with some microcracks can be noted on the wear surface of the Ni-B coating shown in Figure 7a. By comparison, a smooth worn surface with some shallow grooves is shown in Figure 7d for the Ni-B-GO coating. The EDS mapping and spectra shown in Figure 7b and Figure 8a demonstrate that 1.1 wt% Zr transferred from the ZrO_2_ ball was enriched in the wear area of the Ni-B deposit. The presence of GOs impeded the mass transfer between the friction interfaces. The 0.1 wt% Zr detected in the EDS spectra shown in Figure 8b did not form a clear concentration area, as displayed in Figure 7e. According to the above analysis, the main wear mechanism of the coatings changed from the adhesive wear to abrasive wear with the co-deposition of GOs.

The EDS mapping and spectra of oxygen (O) for the wearing surface of the Ni-B alloy and Ni-B-GO composite coating are shown in Figure 7c,f and Figure 8, respectively. For the alloy coating, 2.9 wt% O was mainly concentrated in the plastic deformation area, especially near the cracks, whereas 0.8 wt% O mainly existed in the wear debris stripped from the main structure of the composite. Friction chemistry and friction heat were the two key factors affecting the oxidation reaction in the friction process. For the Ni-B deposit, severe plastic deformation and microcracks caused by cyclic loading constantly generated active surfaces and short-circuit channels for oxidation reactions, thus accelerating the adsorption and diffusion of reactants. GO dispersion in the Ni-B matrix alleviated the friction chemistry by improving the local deformation resistance of the composite. Additionally, the frictional oxidation rate of the composite was reduced with a decrease in friction heat because of the lower COF. In addition, the oxidation product accumulated around the asperities.

### 3.5. Corrosion Resistance

**Figure 9:**
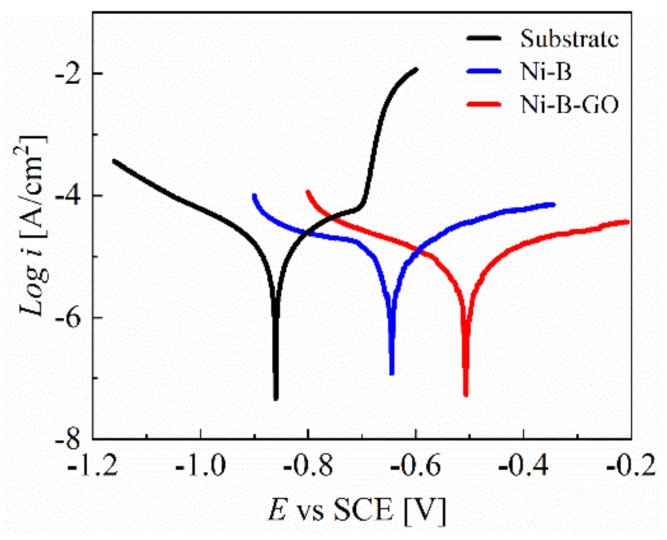
PDP curves of the substrate, Ni−B coating, and Ni−B−GO coating.

PDP curves for the 7075 Al alloy and the coatings were recorded in a 3.5 wt% NaCl corrosive medium, as shown in Figure 9. The PDP curve of the substrate rose sharply at −0.70 V, showcasing the appearance of pitting. However, for the Ni-B and Ni-B-GO coatings, the anodic polarization regions in the measured range showed more stable behavior without the initiation of pitting. The parameters obtained from the Tafel curves are shown in Table 2. A shift in the PDP curve towards a lower *i*_corr_ and higher corrosion potential (*E*_corr_) was observed for Ni-B-GO as compared to the substrate and Ni-B coating, signifying the increased anti-corrosion property of the composite. In addition, the decrease in *CR* also proved that the composite had a stronger resistance to the corrosion medium.

The improvement in the corrosion resistance for the Ni-B-GO coating is mainly ascribed to the compact structure of the composite with a finer crystal grain size [36,43]. During the electrodeposition of the Ni-B matrix on the Al alloy surface, the deposit usually formed various microscopic defects such as microcavities, gaps, and crevices. GOs incorporated into the Ni-B matrix can block the short circuit channel for the absorption and penetration of the corrosive ions [36,45]. As noted previously, the composite deposit shows a compact surface with a finer grain size. The cathode/anode surface ratio is reduced by the finer nanocrystalline structure, which contributes to improving the resistance against localized corrosion [36,46].

**Figure 10:**
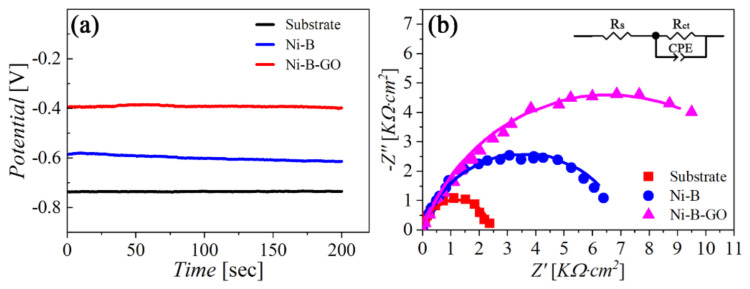
(**a**) OCPT curves and (**b**) Nyquist plots for the substrate, Ni−B coating, and Ni−B−GO coating in 3.5 wt% NaCl solution.

The EIS technique was also applied to evaluate the corrosion behavior of different samples. Figure 10 shows the OCP vs. time (OCPT) curves and Nyquist diagrams for the substrate and the coatings in 3.5 wt% NaCl solution. The composite had the highest OCP value (−0.40 V) compared with the substrate (−0.61 V) and the alloy coating (−0.74 V). A similar trajectory can be observed in Figure 10b from the Nyquist plots of all the samples within the investigated range of frequencies. In addition, the largest capacitive loop corresponding to the composite indicates that the material was highly resistant to corrosion.

To quantitatively analyze the corrosion behavior of working electrodes, we applied an equivalent circuit (EC) to fit the Nyquist curves. As shown in the inset in Figure 10b, the EC model was composed of the solution resistance (*R*_s_), the charge transfer resistance (*R*_ct_), and the constant phase element (CPE). The scattered dots and solid lines in Figure 10b represent the EIS records and the fitting data, respectively. Component parameters of the EC model are tabulated in Table 3. *T* is the capacitance of the CPE, and *P* is a parameter reflecting the similarity between the CPE and an ideal capacitor. It is worth noting that *R*_ct_ represents how difficult it is for a charge to cross the interface between the electrode and the electrolyte during the electrode process. The highest *R*_ct_ value for the Ni-B-GO coating revealed that the composite possessed the best anti-corrosion capability among the samples.

## 4. Conclusions

Ni-B-GO composite coatings were successfully prepared on the surface of 7075 Al alloy using the composite electrodeposition technique. GOs distributed in the composite changed the surface morphology and microstructure of the Ni-B-GO deposit as well as improving its microhardness and resistance to abrasiveness and corrosion. The compact structure of the Ni-B-GO coating with a finer crystal grain resulted in improved corrosion resistance. The enhanced microhardness and wear resistance were mainly attributed to the unique mechanical properties and structure of GOs. Grain refinement caused by the addition of GOs further improved the surface hardness and abrasive performance. Coupled with the conclusion above, GOs incorporated into the Ni-B matrix could enable the use of Ni-B-GO coatings as protective films for Al alloys.

## Figures and Tables

**Table 1:** Composition of the Ni-B electrolyte and deposition parameters [33].

Bath Composition	Concentration (g∙L^−1^)	Depositing Parameters	Values
NiSO_4_∙6H_2_O	240	Temperature (°C)	40
NiCl_2_∙6H_2_O	45	pH	4.8
H_3_BO_3_	30	Current density (A∙dm^−2^)	4
Trimethylamine borane (TMAB)	3	Time (min)	60
Sodium dodecyl sulfate (SDS)	0.01	Magnetic stirring (r∙min^−1^)	300
Saccharin	0.5		

**Table 2:** Tafel curve analysis for Ni-B and Ni-B-GO coatings.

Coating	*E*_corr_ (V)	*i*_corr_ (μA∙cm^−2^)	*CR* (×10^−3^ mm∙y^−1^)
Ni-B	−0.65	27.17	116.64
Ni-B-GO	−0.51	9.13	33.66

**Table 3:** EIS parameters determined for the samples.

Samples	*R*_s_ (Ω∙cm^2^)	*t*	*p*	*R*_ct_ (kΩ∙cm^2^)
Substrate	5.32	4.73 × 10^−5^	0.90	2.42
Ni-B	8.79	6.01 × 10^−5^	0.82	5.03
Ni-B-GO	8.31	6.35 × 10^−5^	0.78	13.12

## Data Availability

Not applicable.
